# Correlation study on the relationship between dyslipidemia and carotid intima-media thickness in patients with diabetes mellitus

**DOI:** 10.12669/pjms.39.3.6866

**Published:** 2023

**Authors:** Hong-jiang Guo, Chen-cong Li, Xiao-yan Bian, Qing Hao

**Affiliations:** 1 Hong-jiang Guo, Department of Physical Examination, The Fourth Hospital of Hebei Medical University, Shijiazhuang 050011, Hebei, P.R. China; 2 Chen-cong Li, Department of Physical Examination, The Fourth Hospital of Hebei Medical University, Shijiazhuang 050011, Hebei, P.R. China; 3 Xiao-yan Bian Health Management Center, The Third Hospital of Hebei Medical University, Shijiazhuang 050051, Hebei, P.R. China; 4 Qing Hao, Department of Physical Examination, The Fourth Hospital of Hebei Medical University, Shijiazhuang 050011, Hebei, P.R. China

**Keywords:** Type-2 diabetes mellitus, Carotid intima-media thickness, Blood lipid, Fasting Plasma glucose, Glycosylated hemoglobin

## Abstract

**Objective:**

To investigate the correlation between dyslipidemia and carotid intima-media thickness (IMT) in patients with diabetes mellitus.

**Methods:**

A descriptive research design was adopted in this study. One hundred and twenty patients with Type-2 diabetes mellitus who were admitted to the physical examination center of The Fourth Hospital of Hebei Medical University from June 2020 to June 2021 for physical examination were recruited to the experimental group. The 120 patients were divided into three groups according to carotid IMT: normal group, thickened group, and plaque group. Forty healthy people who underwent a physical examination during the same period were recruited as the control group. The differences in IMT in various parts of the experimental group and the control group and the differences in blood lipid indexes were compared and analyzed. In addition, the correlation between mean IMT of bilateral common carotid arteries and blood lipid levels in normal, thickened and plaque groups was compared and analyzed.

**Results:**

The intima-media thicknesses of the internal carotid artery and bilateral common carotid arteries of the patients in the experimental group were significantly thicker than those in the healthy control group, the levels of TC, TG and LDL were higher than those in the control group, while the level of HDL was lower than that in the control group, with a statistically significant difference (p=0.00). The levels of fasting plasma glucose (FPG), glycosylated hemoglobin (HbA1c), TG, TC and LDL were positively correlated with the mean IMT of bilateral common carotid arteries (p<0.05), while the level of HDL was negatively correlated with the mean IMT of bilateral common carotid arteries (p<0.05).

**Conclusion:**

Dyslipidemia and glucose metabolism in patients with Type-2 diabetes mellitus have a close bearing on carotid IMT. Clinically, patients with Type-2 diabetes mellitus can be judged by monitoring carotid IMT for dyslipidemia, atherosclerosis and other related complications.

## INTRODUCTION

Type-2 diabetes mellitus is a common metabolic disease in middle-aged and elderly patients.^1^ It is mainly a chronic disease caused by a variety of factors such as heredity and environment, with glucose metabolism disorder as the main manifestation.^2^ With the change in people’s lifestyles, Type-2 diabetes mellitus gradually makes inroads in younger people^3^ and is mainly characterized by hyperglycemia, hyperlipidemia, and the resulting vascular lesions.^4^ It has been considered in studies^5^ that diabetes mellitus is an independent risk factor for cardiovascular and cerebrovascular diseases, and dyslipidemia is the direct cause of atherosclerosis.^6^ Carotid intima-media thickness (IMT) is associated with local lipid accumulation and is a strong predictor of subclinical atherosclerosis.^7^ IMT values are associated with hypercholesterolemia regardless of genetic etiology.^8^ In this clinical study, an in-depth study was conducted on the relationship between dyslipidemia and carotid IMT in patients with type two diabetes mellitus, which is beneficial for clinicians to make early judgments and prevention of diabetes-related complications.

## METHODS

A descriptive research design was adopted in this study. A total of 120 patients with Type-2 diabetes mellitus who were included to the physical examination center of The Fourth Hospital of Hebei Medical University from June 2020 to June 2021 for physical examination were recruited to the experimental group. The 120 patients were divided into three groups according to carotid intima-media thickness (IMT): normal group (MT<1.0 mm), thickened group (1.0 ≤ IMT< 1.5 mm), and plaque group (IMT≥1.5 mm but with no luminal stenosis). An additional 40 healthy people who underwent a physical examination during the same period were recruited as the control group. The study was approved by the Institutional Ethics Committee of The Fourth Hospital of Hebei Medical University (No.:2022KY383; date: March 30, 2022), and written informed consent was obtained from all participants.

### Inclusion criteria:

- Patients aged <70 years old;
- Patients who met the diagnostic criteria for Type-2 diabetes mellitus with a course of disease ≥five years;^9^
- Patients who met the diagnostic criteria of abnormal lipid metabolism;^10^
- Patients who volunteered to participate in the study and signed informed consent;
- Patients who were able to cooperate with the completion of the study;
- Patients who signed the informed consent.

### Exclusion criteria:

- Patients with type one diabetes mellitus;
- Patients with diabetic ketoacidosis or hyperosmolar diabetic coma;
- Patients with poor physical condition or neurasthenia;
- Patients with major organ dysfunctions such as severe heart, pulmonary dysfunction, liver and kidney insufficiency;
- Patients who recently took drugs that affected the study, such as hormones or immunosuppressants;
- Patients who have recently suffered from trauma, surgery, acute or chronic infection and other stress states.

No significant difference was observed in the comparison of general data between the two groups, which was comparable (Table-I).

**Table-I:** Comparative analysis of general data between the experimental group and the control group (*χ̅*±*S*).

Indicators	Experimental group	Control group	t/χ^2^	P
n	120	40		
Age (years old)	62.85±4.83	61.40±5.67	0.56	0.12
Gender (Male, %)	78 (55%)	23 (58.3%)	0.90	0.34
BMI (kg/m^2^)	27.85±4.53	26.57±4.62	0.98	0.33
Hypertension (cases, %)	67 (%)	20 (%)	0.03	0.85
History of hyperlipidemia (cases, %)	74 (41.7%%)	19 (38.3%)	2.47	0.12

p>0.05.

### Laboratory examination indicators:

A total of 160 patients were inclu in the experimental group and the control group. Their venous blood was collected in the morning and on an empty stomach, and the levels of fasting blood glucose (FPG) and glycosylated hemoglobin (HbA1c) were measured with a blood glucose detector. Besides, total cholesterol (TC), triglyceride (TG), low-density lipoprotein (LDL) and low-density lipoprotein (HDL) were detected by an automatic biochemical analyzer. Hypercholesterolemia was defined as serum total cholesterol ≥(5.2 mmol/L) on an empty stomach; (2) IMT detection: Color Doppler ultrasound was used to detect IMT with a probe frequency of 8-14 Hz. The patients were instructed to lie supine with their heads turned to one side to fully expose their necks. The carotid intima-media thickness (IMT) near the distal end of 1.5 cm below the bilateral common carotid artery enlargement was detected, and the average value of the two carotid IMT taken three times was used as the IMT value. According to the diagnostic criteria of IMT: IMT<1.0 mm is considered normal; 1.0 ≤ IMT< 1.5 mm is considered as thickening; IMT≥1.5 mm but with no luminal stenosis is considered arterial plaque.^11^ Based on this, 46 of the 120 patients in the experimental group were included in the normal group, 40 in the thickening group, and 34 in the plaque group.

The differences in IMT in different parts between the experimental group and the control group were compared and analyzed; The differences in TC, TG, LDL, HDL and other blood lipid indexes between the experimental group and the control group were compared and analyzed. The differences of fasting plasma glucose (FPG), glycosylated hemoglobin (HbA1c) and blood lipid levels among the normal group, the thickened group and the plaque group were compared and analyzed. The correlation between mean IMT of bilateral common carotid arteries and TC, TG, LDL, HDL was analyzed.

All data in this study were analyzed with SPSS 20.0 software, and measurement data were expressed as (*χ̅*±*S*). Two independent samples t-test was employed for inter-group data analysis, and c^2^ was used for rate comparison. The correlation between IMT thickness and blood lipid indexes was expressed by the Pearson correlation coefficient, and one-way ANOVA was used to analyze the differences in blood lipid levels among the normal group, the thickened group and the plaque group. P<0.05 indicates a statistically significant difference.

## RESULTS

The comparative analysis of IMT in various parts between the experimental group and the control group (Table-II) showed that the intima-media thicknesses of the internal carotid artery and bilateral common carotid arteries of the patients in the experimental group were significantly thicker than those in the healthy control group, with a statistically significant difference (p=0.00).

**Table-II:** Differential analysis of IMT in different parts of patients in the experimental group and the control group (*χ̅*±*S*).

Group	n	Left internal carotid artery	Right internal carotid artery	Left common carotid artery	Right common carotid artery
Experimental group	120	1.35±0.12	1.37±0.23	1.31±0.21	1.38±0.21
Control group	40	1.22±0.11	1.24±0.17	1.20±0.19	1.23±0.20
t		11.18	6.07	10.14	8.71
p		0.00	0.00	0.00	0.00

p<0.05

The comparative analysis of TC, TG, LDL, HDL and other blood lipid indexes between the experimental group and the control group (Table-III) showed that the levels of TC, TG and LDL in the experimental group were higher than those in the control group, with a statistically significant difference (p=0.00), while the level of HDL was lower than that in the control group, with a statistically significant difference (p=0.00). In the experimental group, the levels of FPG, HbA1c, TG, TC and LDL increased with the thickness of IMT, with a statistically significant difference among the groups (p<0.05), while the level of HLD decreased with the increase of IMT thickness, with a statistically significant difference among the groups (p=0.01) (Table-IV).

**Table-III:** Differential analysis of blood lipid indexes between the experimental group and the control group (*χ̅*±*S*).

Group	n	TC (mmol/L)	TG (mmol/L)	LDL (mmol/L)	HDL (mmol/L)
Experimental group	120	4.36±0.72	1.54±0.32	2.73±0.45	1.23±0.12
Control group	40	3.31±0.68	1.05±0.27	2.43±0.61	1.64±0.20
t		6.55	8.70	3.32	15.60
p		0.00	0.00	0.00	0.00

p<0.05

**Table-IV:** Differentiation analysis of blood lipids and levels of FPG and HbA1c among the normal group, the thickened group and the plaque group (*χ̅*±*S*).

Group	n	FPG (mmol/L)	HbA1c (%)	TC (mmol/L)	TG (mmol/L)	LDL (mmol/L)	HDL (mmol/L)
Normal group	46	6.73±1.21	9.83±2.61	4.03±0.83	1.27±0.31	2.58±0.23	1.59±0.76
Thickened group	40	8.45±1.53	11.55±2.59	4.36±0.44	1.65±0.23	2.73±0.46	1.32±0.49
Plaque group	34	9.07±1.38	13.06±2.27	4.67±0.74	1.97±0.52	2.90±0.42	1.15±0.31
F		2.39	3.51	2.50	3.76	2.16	2.58
p		0.02	0.00	0.01	0.00	0.03	0.01

p<0.05.

Correlation analysis showed that the levels of FPG, HbA1c, TG, TC and LDL were positively correlated with the mean IMT of bilateral common carotid arteries (p<0.05), while the level of HDL was negatively correlated with the mean IMT of bilateral common carotid arteries (p<0.05) (Table-V).

**Table-V:** Correlation analysis between the mean IMT of bilateral common carotid arteries and the levels of blood lipids, FPG and HbA1c (*χ̅*±*S*)n=60.

Variables	r	p
TC	0.78	0.00
TG	0.70	0.00
LDL	0.65	0.00
HDL	-0.67	0.00
FPG	0.59	0.02
HbA1c	0.62	0.01

p<0.05

## DISCUSSION

It was confirmed in our study that the intima-media thicknesses of the internal carotid artery and bilateral common carotid arteries in patients with T2DM were significantly thicker than those in the healthy control group, with a statistically significant difference (p=0.00). The levels of TC, TG and LDL in patients with T2DM were higher than those in the control group, with a statistically significant difference (p=0.00), while the level of HDL was lower than that in the control group, with a statistically significant difference (p=0.00). It was confirmed by Purnamasari^12^ that the mean IMT of first-degree relatives (FDR) of hyperlipidemia was higher than that of non-FDR, and significant differences were maintained between subjects. The IMT of FDR in young adults with T2DM is thicker than that in the non-FDR population. These were similar to the results of our study.

Diabetes mellitus is a common clinical endocrine and metabolic disease with genetic susceptibility, which is caused by changes in environmental, genetic and other comprehensive factors.^13^ Globally, with the aggravation of population aging and the change in people’s lifestyles, Type-2 diabetes mellitus (T2DM) is seen to have an increasing incidence rate year by year, especially in developing countries, showing an epidemic trend.^14^ All in all, the threat that diabetes mellitus poses to humans is approaching that of vascular disease and cancer. As a chronic disease, diabetes mellitus is mainly characterized by glucose and lipid metabolism disorders and vascular lesions,^15^ among which atherosclerosis is the main cause of death in patients with T2DM. Carotid intima-media thickening is an early manifestation of atherosclerosis, which has a close bearing on a variety of vascular risk factors. It is a satisfactory indicator of arteriosclerosis disease and is closely related to the occurrence of macrovascular disease in the early stage of diabetes mellitus.^16^ Carotid intima-media thickening represents early atherosclerosis, while plaques and luminal stenosis represent advanced atherosclerosis. Through descriptive analysis, this study found that abnormal lipid and glucose metabolism in Type-2 diabetes patients was closely related to carotid IMT.

The carotid artery is the most commonly used part for ultrasonic detection of superficial atherosclerosis due to its superficial location and ease to be identified by ultrasound^17^ Carotid intima-media thickness and plaque are closely related to many cardiovascular and cerebrovascular diseases. Ultrasound detection of carotid intima-media and plaque is a non-invasive examination for evaluating atherosclerosis, which has been widely used in clinics.^18^ In terms of the mechanism of atherosclerosis, lipid deposits are deposited under the intima after injury of the arterial intima, which leads to atherosclerosis and calcification of the vascular wall as well as decreased vascular compliance of the vascular wall^19^ Such a change often causes serious damage to the heart, brain, kidney and other parenchymal organs with rich blood circulation. By detecting the thickness of carotid intima-media, early detection, intervention and treatment of vascular complications of Type-2 diabetes mellitus can be carried out to ameliorate the quality of life of patients and reduce the incidence of cardiovascular and cerebrovascular diseases.^20^ It was considered by Akazawa et al.^21^ that IMT provides a superior first-line screening method for detecting cardiovascular and cerebrovascular complications in patients with asymptomatic diabetes mellitus. Ultrasound carotid artery imaging may contribute to the management of type two diabetes mellitus.^22^

According to Harmer et al.^23^, IMT is a marker of subclinical atherosclerosis. Creatinine, total cholesterol, LDL cholesterol and triglyceride are risk factors promoting atherosclerosis. Bulut et al.^24^ believed that systolic blood pressure (SBP), total cholesterol and high-density lipoprotein cholesterol levels were independent risk factors affecting IMT (p<0.001). Bouillet^19^ and colleagues argued that continuous abnormal lipid metabolism increases the potential risk of atherosclerosis, and that carotid intima-media thickness (a marker of early atherosclerosis) is correlated with various indexes of blood lipid. In our study, the levels of FPG, HbA1c, TG, TC and LDL increased with the thickness of IMT (p<0.05), while the level of HLD decreased with the increase of IMT thickness (p=0.01). The levels of FPG, HbA1c, TG, TC and LDL were positively correlated with the mean IMT of bilateral common carotid arteries (p<0.05), while the level of HDL was negatively correlated with the mean IMT of bilateral common carotid arteries (p<0.05). These results were consistent with the conclusions of previous studies. These findings provide a clinical basis for the clinical significance of using ultrasound to monitor carotid IMT in patients with type two diabetes mellitus.

### Limitations:

Nonetheless, deficiencies remain in this study: fewer samples were recruited and follow-up was not included. In response to this, the sample size will be further expanded and follow-up contents will be increased in the future clinical work. In this way, the value of carotid intima-media thickness and blood lipid indexes for prognosis and long-term complication evaluation can be further confirmed, so as to benefit more patients.

## CONCLUSION

***To put it in a nutshell:*** Dyslipidemia and glucose metabolism in patients with Type-2 diabetes mellitus have a close bearing on carotid IMT. Clinically, patients with Type-2 diabetes mellitus can be judged by monitoring carotid IMT for dyslipidemia, atherosclerosis and other related complications Ultrasound detection of IMT is an effective non-invasive method.

### Authors’ Contributions:

**Hong-jiang Guo** and **Chen-chen Li:** Designed this study and prepared this manuscript, are responsible and accountable for the accuracy and integrity of the work.

**Xiao-yan Bian:** Collected and analyzed clinical data.

**Qing Hao:** participated in acquisition, analysis, and draft the manuscript. All authors read and approved the final manuscript.
